# ZIP Code and GIS Studies: Kouznetsova et al. Respond

**DOI:** 10.1289/ehp.10840R

**Published:** 2008-01

**Authors:** Maria Kouznetsova, Xiaoyu Huang, Jing Ma, Lawrence Lessner, David O. Carpenter

**Affiliations:** Institute for Health and the Environment, University at Albany, SUNY, Rensselaer, New York, E-mail: carpent@uamail.albany.edu

There is strong evidence that exposure to persistent organic pollutants (POPs), including polychlorinated biphenyls (PCBs), is associated with an increased risk of diabetes, and that evidence does not come only from studies of residential proximity to waste sites. In our studies in an adult Native-American population, individuals in the top tertile of serum PCBs showed a 3.9-fold [95% confidence interval (CI), 1.5–10.6] elevated risk of diabetes after adjustment for age, body mass index, smoking, sex, and serum lipid levels (Codru et al. 2007). Diabetes was defined as either taking prescription antidiabetes medication or having a fasting glucose level of > 125 mg/dL. In another study, Lee et al. (2006), using NHANES (National Health and Nutrition Examination Survey) data, found a dose-dependent relationship between prevalence of diabetes and serum concentrations of six different organochlorine compounds, including one PCB. Other studies reporting this relationship are cited in our recent article (Codru et al. 2007).

The occupational studies listed by Golden and Schell do not demonstrate a relationship with diabetes. However, absence of evidence does not mean evidence of absence. Most occupational studies have poor exposure assessment and short follow-up periods, and are compromised by the healthy-worker effect.

Studies such as ours (Kouznetsova et al. 2007) do, of course, have limitations, in that we do not have personal information of exposure to individuals beyond their ZIP code of residence. But they also have strengths, especially in the very large numbers of hospitalizations and the uniformity of the data-collection system in New York. We recognize the limitations, which we discussed extensively in our article. Although it is difficult to control for all confounders in investigations such as this, they are hypothesis generating and should lead to studies where exposure can be better assessed. This we have done. Our parallel study (Codru et al. 2007), in which we assessed exposure by measuring serum PCBs and fasting glucose, provided extremely strong support for the conclusion of Kouznetsova et al. (2007): that simply living near a PCB-contaminated site (in this case the Hudson River) poses a risk of both exposure and disease.

We have also reported an elevated rate of hospitalization for cardiovascular disease and heart attacks among individuals living in ZIP codes containing waste sites contaminated with POPs, especially along the Hudson River (Sergeev and Carpenter 2005). Also, in another study with excellent exposure assessment, we demonstrated that elevated exposure to PCBs leads to elevated levels of serum lipids and heart disease in a human population (Goncharov et al. 2007).

Golden and Schell actually have two arguments, each fallacious. First, they discount our evidence that residential proximity to hazardous waste sites leads to disease, arguing that some others have not demonstrated elevated serum PCB levels. The above observations show that this is not so. Golden and Schell’s last sentence basically accepts the evidence that “increased incidence of diabetes [is] associated with environmental exposure to PCBs,” but then they argue that this relationship is a consequence of diabetes-related metabolic perturbations. You really cannot have it both ways. We have demonstrated that residence near the Hudson River (where average income is higher and there is less smoking, more exercise, and better diet than in the rest of New York State) is associated with increased rates of hospitalization for not only diabetes and heart disease but also hypertension (Huang et al. 2006), stroke (Shcherbatykh et al. 2005), and chronic respiratory disease (Kudyakov et al. 2004). These associations cannot be explained away by “diabetes-related metabolic perturbations.”

The mechanisms responsible for the relationship between PCB exposure and these multiple chronic diseases are not certain, but it is likely that they result from secondary to posttranscriptional gene regulation. The studies of Adeeko et al. (2002) and Vezina et al. (2004) demonstrate that a very large number of diverse genes show altered expression upon exposure to POPs. There is still a lot that we do not know, but it is very clear that these chemicals are dangerous compounds and that exposure to them is associated with an elevated risk of a variety of chronic human diseases.

## References

[b1-ehp0116-a0018b] Adeeko A, Li D, Doucet J, Cooke GM, Trasler JM, Robaire B (2002). Gestational exposure to persistent organic pollutants: maternal liver residues, pregnancy outcome, and effects on hepatic gene expression profiles in the dam and fetus. Toxicol Sci.

[b2-ehp0116-a0018b] Codru N, Schymura MJ, Negoita S, Rej R, Carpenter DO, Akwesasne Task Force on the Environment (2007). Diabetes in relation to serum levels of polychlorinated biphenyls and chlorinated pesticides in adult Native Americans. Environ Health Perspect.

[b3-ehp0116-a0018b] Goncharov A, Haase RF, Santiago-Rivera A, Morse G, McCaffrey RJ, Akwesasne Task Force on the Environment (2007). High serum PCBs are associated with elevation of serum lipids and cardiovascular diseases in a Native American population. Environ Res.

[b4-ehp0116-a0018b] Huang X, Lessner L, Carpenter DO (2006). Exposure to persistent organic pollutants and hypertensive disease. Environ Res.

[b5-ehp0116-a0018b] Kouznetsova M, Huang X, Ma J, Lessner L, Carpenter DO (2007). Increased rate of hospitalization for diabetes and residential proximity of hazardous waste sites. Environ Health Perspect.

[b6-ehp0116-a0018b] Kudyakov R, Baibergenova A, Zdeb M, Carpenter DO (2004). Respiratory disease in relation to patient residence near to hazardous waste sites. Environ Toxicol Pharmacol.

[b7-ehp0116-a0018b] Lee DH, Lee IK, Song K, Steffes M, Toscano W, Baker BA (2006). A strong dose-response relation between serum concentrations of persistent organic pollutants and diabetes. Diabetes Care.

[b8-ehp0116-a0018b] Sergeev A, Carpenter DO (2005). Hospitalization rates for coronary heart disease in relation to residence near areas contaminated with persistent organic pollutants and other pollutants. Environ Health Perspect.

[b9-ehp0116-a0018b] Shcherbatykh I, Huang X, Lessner L, Carpenter DO (2005). Hazardous waste sites and stroke in New York State. Environ Health.

[b10-ehp0116-a0018b] Vezina CM, Walker NJ, Olson JR (2004). Subchronic exposure to TCDD, PeCDF, PCB126, and PCB153: effect on hepatic gene expression. Environ Health Perspect.

